# The diseased kidney: aging and senescent immunology

**DOI:** 10.1186/s12979-022-00313-9

**Published:** 2022-11-16

**Authors:** Mingxuan Chi, Zijun Tian, Kuai Ma, Yunlong Li, Li Wang, Moussa Ide Nasser, Chi Liu

**Affiliations:** 1 Department of Nephrology, Sichuan Academy of Medical Science and Sichuan Provincial People’s Hospital, Sichuan Renal Disease Clinical Research Center, University of Electronic Science and Technology of China, Chengdu, China; 2 Chinese Academy of Sciences Sichuan Translational Medicine Research Hospital, Chengdu, 610072 China; 3 Department of Anesthesiology, Women’s Hospital of Nanjing Medical University, Nanjing Maternity and Child Health Care Hospital, Nanjing, China; 4 Department of Nephrology, Osaka University Graduate School of Medicine, Osaka, Japan; 5 Department of Urology, Sichuan Cancer Hospital & Institute, Sichuan Cancer Center, School of Medicine, University of Electronic Science and Technology of China, Chengdu, China; 6 Guangdong Cardiovascular Institute, Guangdong Provincial People’s Hospital, Guangdong Academy of Medical Sciences, Guangzhou, 510100 Guangdong China; 7 Renal Department and Nephrology Institute, Sichuan Provincial People’s Hospital, School of Medicine, University of Electronic Science and Technology of China, Clinical Research Center for Kidney Diseases, Chengdu, Sichuan China

**Keywords:** Immunosenescence, Aging, Kidney diseases, Inflammaging, Immunotherapy

## Abstract

Immunosenescence is the deterioration of the innate and adaptive immune systems associated with aging and is primarily characterized by a reduction in T cell production and accumulation of atypical subsets. Age-related immunological dysfunction leads to impaired immune protection and persistent low-grade chronic inflammation, resulting in a decreased vaccination response and increased vulnerability to infection, cancer, cardiovascular disease, and autoimmune disease in the elderly. As the elderly constitute a growing proportion of the population with renal disease, immunosenescence is a normal aging process that is prevalent among older people. In addition, immunosenescence seems to be more pronounced in patients with kidney diseases than in healthy controls, as shown by severe chronic inflammation, accumulation of immune cells with the senescent phenotype (CD28^−^ T cells, CD14^+^CD16^+^ monocytes), and proinflammatory cytokine production. Immunosenescence inhibits immunological clearance and renal tissue regeneration, thereby increasing the risk of permanent renal damage, infection, and cardiovascular events in patients with kidney disease, lowering the prognosis, and even influencing the efficacy of renal replacement treatment. Biological drugs (senomorphics and senolytics) target the aging immune system and exert renoprotective effects. This review aims to emphasize the features of immunosenescence and its influence on kidney diseases and immunotherapy, highlighting the future directions of kidney disease treatment using senescence-focused techniques.

## Introduction

Advances in medical technology have significantly enhanced life expectancy. The worldwide population aged 60 years or older is expected to quadruple by 2050, representing 22% of the global population [1]. In response to the worldwide issues of age-related diseases caused by an aging population, the study of aging has gained growing interest. Intrinsic and extrinsic factors shape the immune system and profoundly change with aging. In other words, the immune response is age dependent [2]. In the 1960s, the gerontologist Roy Walford first introduced the term “immunosenescence”; his landmark book, “The Immunologic Theory of Aging,” laid the foundation for many advanced studies about immunity and aging [3]. The change of cellular phenotype and the immune system composition, termed “immunosenescence” presents two opposing characteristics: increased susceptibility to infectious disease and persistent systemic inflammation. Consequently, the risk of many degenerative diseases [4], especially neurodegeneration [5], cancer [6], cardiovascular [7], and autoimmune diseases [8], is higher in people with immunosenescence. Therefore, preserving immune function may reduce disease incidence and prolong life. Numerous studies on the underlying mechanisms of age-related immune decline have laid the groundwork for approaches to cancer and chronic diseases. Immune checkpoint blockade therapies, such as anti-programmed death 1 (PD-1) and anti-cytotoxic T-lymphocyte-associated protein 4 (CTLA-4), have provided new hope for the treatment of hematological malignancies [9]. Cryopreservation of immune cells and immune cell modification techniques play positive roles in treating senile tumors [10]. T cells are genetically modified to express the chimeric antigen receptor for a specific cell surface antigen to identify and kill tumor cells. The emerging use of senolytics and senomorphics has been investigated to delay aging and treat chronic diseases in humans [11].

Kidney disease has been recognized as a major public health burden over the past decade. The prevalence of chronic kidney disease (CKD) exceeds 10% and is > 50% in high-risk subpopulations [12]. Aging kidneys undergo structural and functional alterations due to the complex interplay between hereditary and environmental changes and cellular dysfunction. Renal structural abnormalities include reduced nephron size and number, tubular interstitial fibrosis, renal capsule fibrosis, glomerulosclerosis, thickening of the glomerular basement membrane, increased glomerulosclerosis, and tubular atrophy. Additionally, renal functional changes manifest as glomerular filtration rate (GFR) decline, accumulation of toxins, and urine and blood component changes [13]. Progressive loss of kidney function ultimately leads to end-stage renal disease (ESRD), characterized by a series of systemic disorders including electrolyte disorders, metabolic dysregulation, and acidosis, also known as uremia, which affects almost all organs in the body.

Cellular senescence is a long-lasting cell cycle arrest induced by replicative senescence (chronological aging) and premature senescence (organ injury) [14], which is suggested to be a hallmark of aging and contributes to chronic diseases. Senescent cells secrete a specific senescence-associated secretory phenotype (SASP), which mainly includes a variety of proinflammatory cytokines (e.g., interleukin (IL)-1α, IL-1β, IL-6, and IL-8), growth factors (e.g., hepatocyte growth factor, transforming growth factor-β, and granulocyte-macrophage colony-stimulating factor), chemokines [e.g., chemokine (C-X-C motif) ligand (CXCL)-1/3 and CXCL-10], and matrix remodeling enzymes (e.g., metalloproteinases) [15]. Renal senescence occurs when senescent cells accumulate in the kidneys and chronic low-grade inflammation is induced. Chronic inflammation impedes intrinsic tissue regeneration and exacerbates renal damage, thus increasing the vulnerability of older adults to kidney disease and reducing their renal regenerative potential.

Age-related changes in the immune system associated with aging play an essential role in the pathogenesis of kidney diseases. Consequently, it is essential to understand the interactions between immunosenescence and renal disorders. Numerous investigations of the underlying processes of age-related alterations in the immune system have paved the way for novel treatment approaches for kidney disorders. This review discusses the recent molecular research on immunosenescence, focusing on innate and adaptive immune cell changes. We will also examine immunosenescence in kidney diseases, evaluate current immunotherapies that target age-related immune system abnormalities, and assess their potential applications in kidney diseases.

### Immunosenescence of innate immune response cells

#### Natural killer (NK) cells

NK cells are key cells in the innate immune system for recognizing and clearing viral infections and abnormal cells (including tumor cells). Recent research has shown that preserving NK cell features in older adults may contribute to longevity and successful aging. Based on the expression levels of the surface molecules CD56 and CD16, NK cells can be divided into two subgroups. CD56^bright^ cells are an immature subset that shows high proliferative activity and the ability to produce a range of cytokines, such as interferon (IFN)-γ, tumor necrosis factor (TNF)-β, and IL-10, and chemokines, such as RANTES and macrophage inflammatory protein-1α. In contrast, CD56^dim^ cells are a mature subset that show high cytotoxic activity and a lower ability to produce cytokines [16]. The peripheral CD56^bright^ NK cells in the elderly is decreased, which may be caused by the degeneration of hematopoietic stem cells (HSCs) in bone marrow (where the NK cells are derived) of aging individuals [17].

Meanwhile, the number of CD56^dim^ NK cell subsets is more in man than that of women [18]. Although the overall cytotoxicity of NK cells did not decrease significantly in healthy older adults, the activity of each NK cell was reduced compared to that in young adults. Extensive phenotypic and functional analysis of NK cells from healthy subjects revealed that NK cells in cord blood displayed specific features, including poor expression of killer immunoglobulin-like receptors and leukocyte immunoglobulin-like receptor-1/immunoglobulin-like transcript receptor-2 (LIR-1/ILT-2), and high expression of both NKG2A and IFN-γ. In contrast, NK cells from older subjects showed a specific increase in LIR-1/ILT-2 levels. The ability to produce IFN-γ was modestly impaired in NK cells from older participants. Importantly, they also observed a perfect recovery of NK cell function in very old subjects following IL2-activation [19]. The expression of natural cytotoxic receptors, such as NKp30 and NKp46, on the surface of NK cells is decreased in the elderly, while both receptors are expressed at high levels in the young [20].

#### Dendritic cells

Dendritic cells (DCs) are the most potent antigen-presenting cells (APC) with TLRs on their surfaces [21]. Upon antigenic stimulation, DCs capture, process, and present pathogen antigens through major histocompatibility complex II (MHC II) and release cytokines, thus priming naïve T cells and initiating adaptive immunity. DCs have been long focused in immunotherapy as a bridge between innate and adaptive immunities.

The myeloid (mDC), plasmacytoid (pDC), and follicular (fDC) dendritic cell subsets are distinct subsets with particular roles. Several investigations have demonstrated age-related impairments in DCs antigen presentation and T-cell activation in the elderly. The production of type I and III IFNs, which enhance the coordination of innate and acquired immune responses, is reduced in elderly pDCs [22]. The activity of PI3 kinase is markedly diminished in the mDCs of aged donors, resulting in altered phagocytic activity and migration in response to inflammatory stimuli. Moreover, mDCs demonstrate abnormal inflammatory cytokine production (IL-6 and TNF-α) due to the overexpression of nuclear factor-kappa B (NF-κB) in elderly adults [23]. The immune response to autoantigens is also enhanced, leading to decreased immune tolerance and chronic inflammation in the elderly population.

Antigen presentation to naïve B cells and the formation of germinal centers by fDCs with highly expressed FcγRII receptors play a critical role in stimulating robust humoral immunity in response to infection or vaccination [24]. Defects in germinal center formation with impaired immunological memory have been observed in aged mice. Age-related decline in FcγRII receptor expression is implicated in diminished antigen retention and presentation ability, leading to a decline in B cell activation and antibody production [25].

#### Monocytes and macrophages

Monocytes express a broad range of pattern recognition receptors and play a crucial role in the innate immune response. Monocytes are activated upon pathogen stimulation via TLRs, and subsequently secrete proinflammatory cytokines and present antigens, exerting various immune effector functions. Human monocytes are identified by the expression of CD14 and CD16 on their cell surface. Classical, intermediate, and non-classical monocytes are defined as CD14^+^CD16^−^, CD14^+^CD16^+^, and CD14^−^CD16^+^, respectively [26]. CD16^+^ monocytes present a more senescent phenotype with shorter telomeres and an increased inflammatory potential [27]. No significant differences were observed in the number of circulating monocytes in older adults, whereas the proportion of CD16^+^ monocytes was higher than that in young adults. Macrophages are tissue-resident cells that are known to phagocytose and initiate an inflammatory signaling cascade. Although inflammation is important for insult defense, it is detrimental to the immune system. Monocytes and macrophages are believed to be central components facilitating low-grade chronic inflammation during immunosenescence [28]. Macrophages are recognized as two different phenotypes, classical (M1) and alternative (M2), based on their different inflammatory responses and cytokine release, including TNFα, IL-1β, and IL-12 [29]. M2 monocytes are labeled as anti-inflammatory monocytes and show an obvious age-related reduction in healthy elderly individuals [30]. A larger monocyte population exists in the elderly with impaired TLR expression and biological responses [31]. Thus, when stimulated with TLR stimuli, they reduce proinflammatory cytokine production (such as IL-1β and IFN-γ) compared to young cells [32]. This may help to elucidate innate immune defects in older people. Aged macrophages produce more cyclooxygenase 2 and subsequently prostaglandin 2, which is correlated with increased expression of inflammatory cytokines, such as TNFα and IL-6.

### Immunosenescence of adaptive immune response cells

**Fig. 1:**
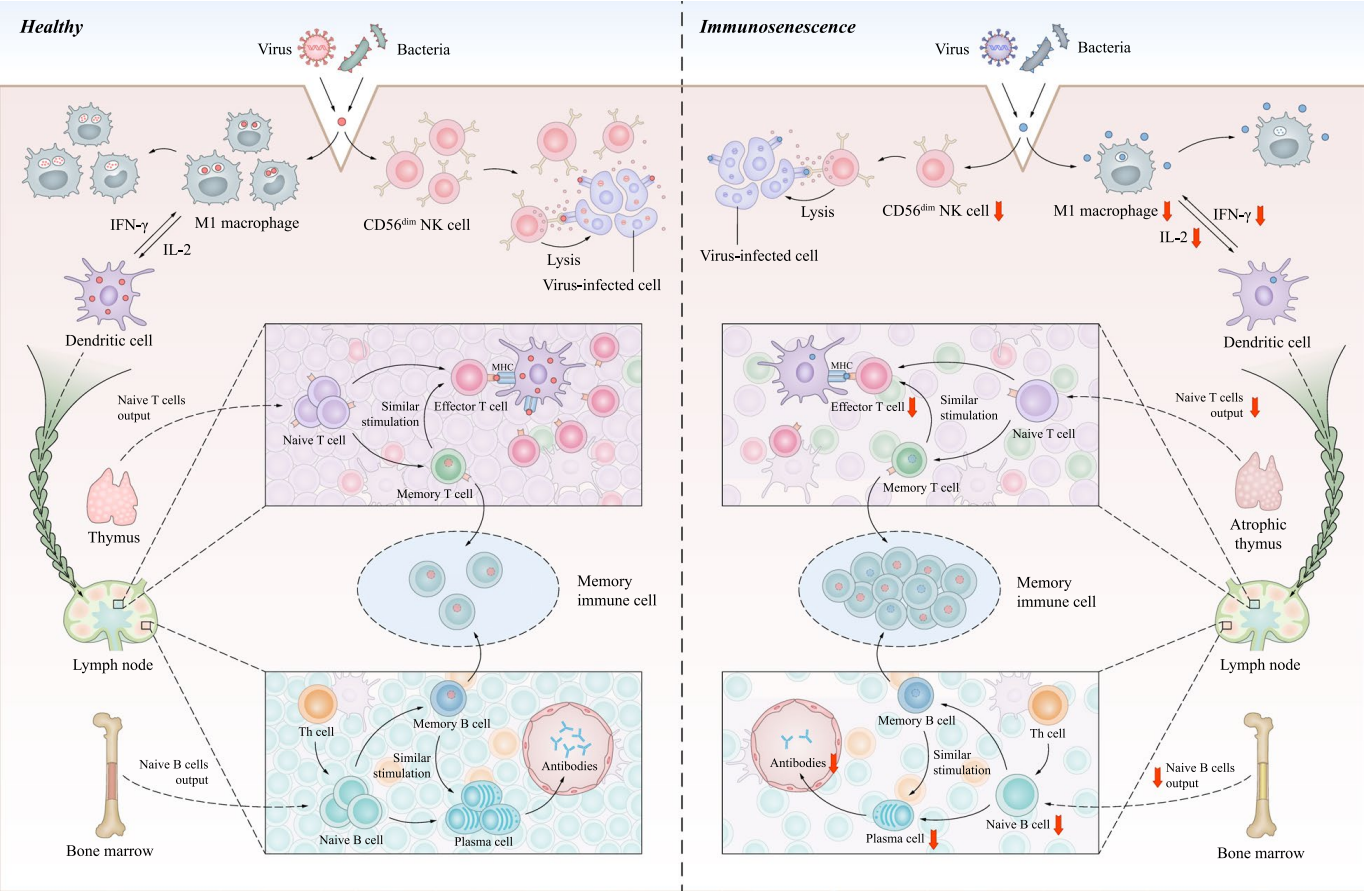
Characteristics of immunosenescence in innate and adaptive immunity. Many immune cell subpopulations are altered during immunosenescence. The mature CD56^dim^ natural killer (NK) cell subsets with high cytotoxic activity are decreased, diminishing the ability to recognize and clear viral infection and tumor cells. Antigen processing and presentation capabilities of macrophages and APCs are also diminished. Declined naïve T cells output of the thymus and decreased antigen stimulation presented by APCs lead to declining T cell response. Fewer naïve B cells are stimulated and transform into plasma cells, thus humoral immunity is impaired. However, the number of memory T and B cells increases

T and B cells are essential for adaptive immunological responses to antigens. Lifetime pathogen exposure is associated with an age-related decline in these immune cells, which is characterized by a reduced output of naïve T/B cells, abnormal phenotype of mature cells, and accumulation of memory cells. These changes in adaptive immune cells result in insufficient immune response to new antigens in older individuals (Fig. 1).

#### T cells

The thymus provides a variety of microenvironments that support the development and export of naïve T-cells to the periphery [33]. Since puberty, the thymus gland undergoes gradual involution as sex hormones increase, decreasing the T-cell repertoire to new antigens, which plays a crucial role in T cell senescence. In addition, reducing IL-7 levels and oxidative stress damage contribute to the onset of thymic atrophy [34–37]. In naïve CD4^+^ T cells, decreased expression of miR-181A promotes the activity of dual-specific phosphatase 6 (DUSP6), thereby contributing to the desensitization of T cell receptors (TCRs) and contraction of TCR diversity [38]. T cells are repeatedly triggered by the same antigens throughout the lifelong chronic antigen load, creating a population of late-differentiated oligoclonal effector memory T cells and reducing the naive T cell reservoir [39]. The adaptive immune response to new antigens is decreased in aged adults due to the shrinkage of naive T cell reservoirs and expansion of immunological memory for previously encountered infections. Moreover, senescent T lymphocytes have subsets with abnormal phenotypes and abnormal expression of cell-surface molecules. After a primary infection, human cytomegalovirus (HCMV) resides in the body and periodically activates the immune system. The accumulation of terminally differentiated HCMV-specific CD8+ T cells and inversion of the CD4+/CD8+ T cell ratio are associated with HCMV seropositivity in the elderly (> 80 years) [40, 41]. Senescent T cells maintain low expression levels of the necessary co-stimulatory molecule CD27/CD28 but upregulate inhibitory receptors on the cell surface, including PD-1 and CTLA-4. CD28^−^ T cells are resistant to apoptosis and less susceptible to modulation by regulatory T cells. Functionally, CD28^−^ T cells exhibit a high cytotoxic trait and express cytotoxic effector molecules (e.g., granase B and perforin) and NK cell receptors, culpable CD28^−^ T cells in the pathogenesis of various age-related diseases [42, 43]. Increased senescent T cells express negative signaling receptors, such as PD-1, which suppresses the T cell response. Interestingly, PD-1 blockade in the elderly partially restores the functional competence of T-cells [44]. Senescent T cells also express CD57 and killer cell lectin-like receptor subfamily G member 1, with high cytotoxic potential [45].

#### B cells

Age also affects the quantity and variety of B cells as the number of naïve B cells decreases, while that of effector B cells increases. In the peripheral blood, immunoglobulins generated by naïve B cells (IgD, IgM) are replaced by those produced by memory B cells (IgG and IgA) [46]. Additionally, the immunological response to foreign antigens and the ability to reorganize immunoglobulin genes are diminished. Reduced E2A gene expression in activated B cells of the elderly diminishes the induction of E47 (a class I basic helix-loop-helix protein produced by the E2A gene), thereby diminishing antibody avidity and antibody-mediated protection [47, 48]. The memory/effector T cells show reduced expression of CD40L and reduced B cell synthesis [49].

### Inflammaging

The state of prolonged, sterile, low-grade inflammation, namely inflammaging, is almost universally present in the elderly [50]. While acute inflammation is regarded as a protective process against harmful pathogens, persistent unresolved inflammation could be disadvantageous. Used to be considered as a part of “immunosenescence,” inflammaging should be regarded as a state different from but closely interacting with immunosenescence. Inflammaging contributes to changes in cellular phenotype and immune system composition, reducing endurance to different antigenic stressors in aging individuals. Both inflammaging and immunosenescence are common mechanisms of age-related decline and diseases, including type 2 diabetes and cardiovascular diseases [51]. In addition, persistent low-grade infection contributes to lineage skewing of HSCs toward myeloid progenitors, and thus the potential of HSCs to replenish lymphoid lineage cells is compromised [52]. Lifelong body adaptation to antigenic load and stress exposure can result in the remodeling of both innate and adaptive immunity, which is globally regarded as immunosenescence.

This state of chronic inflammation is associated with the continuous activation of macrophages via pattern recognition receptors [Toll-like receptors (TLRs). nucleotide-binding oligomerization domain (NOD)-like receptors] interact with damage-associated molecular patterns (DAMPs)/pathogen-associated molecular pattern (PAMPs)/metabolism-associated molecular patterns (MAMPs) that initiate the proinflammatory cascade [53]. The specific changes in senescent microbiota and gut barrier injury in the elderly are great sources of disease-associated pathobionts, which are associated with an increased local and systemic inflammatory status, sustaining inflammaging and dialogue with other organs, particularly the kidneys [54]. Recent reports have shown overexpression of TLRs in multiple kidney diseases, including autoimmune renal diseases, vasculitis, acute kidney injury (AKI), and kidney transplant rejection. TLRs signals activate the myeloid differentiation primary response protein 88 (MyD88)/TIR-domain-containing adapter-inducing interferon-β, and then activate NF-κB target genes, a transcription factor that regulates inflammatory and oxidative stress pathways [55, 56]. Excessive activation of NF-κB leads to an asymptomatic inflammatory tone (IL-1, IL-6, IL-15, IL-18, TNF, and C-reactive protein), renal fibrosis, and CKD progression [57–60]. Danger signals activate NOD-like receptors, which can form multi-protein complexes or inflammasomes, promote the cleavage of pro-caspase 1 into active caspase-1, and subsequently activate proinflammatory cytokines, including IL-18 and IL-1β [61]. In experimental models, inflammasomes and inflammasome-related genes, particularly NOD-, LRR-, and pyrin domain-containing 3, contribute to the pathogenesis of a wide range of chronic kidney disease, acute kidney injury, and diabetic kidney disease via canonical and non-canonical mechanisms that regulate inflammation, pyroptosis, apoptosis, and fibrosis [62]. During the inflammatory process, cells of innate immunity, such as phagocytic cells, produce high amounts of reactive oxygen species (ROS) to eliminate foreign agents. High ROS levels may contribute to the oxidative damage associated with many diseases and aging. Moreover, oxidant components can act as intracellular secondary messengers during the inflammatory response [63]. Overproduction of oxidative stress can induce an inflammatory response, thus establishing a vicious cycle that fuels immunosenescence. As a result of age-related oxidative injury, senescent cells lose some of their capacity to regulate their own redox and inflammatory balance, which is the basis of immunosenescence, namely “oxi-inflamm-aging’ [64].

### Immunosenescence and kidney diseases

#### Immunosenescence in acute kidney injury

AKI is a life-threatening clinical condition with a high incidence and mortality rate (16–50%) [65]. AKI is associated with an abrupt decrease in kidney function caused by pathogenic conditions such as ischemia and toxic stimuli. AKI is considered a self-limiting syndrome, with most survivors regaining renal function after damage. However, 5% of AKI survivors live with persistent renal impairment and require continuous renal replacement therapy. In older patients, this proportion may be as high as 16%. Patients with severe AKI can develop interstitial fibrosis, CKD, and ESRD later in life [66, 67]. Previous research has reported that renal injury could induce premature aging in the kidneys, suggesting that these changes increase the propensity to develop subsequent progressive CKD.

**Fig. 2:**
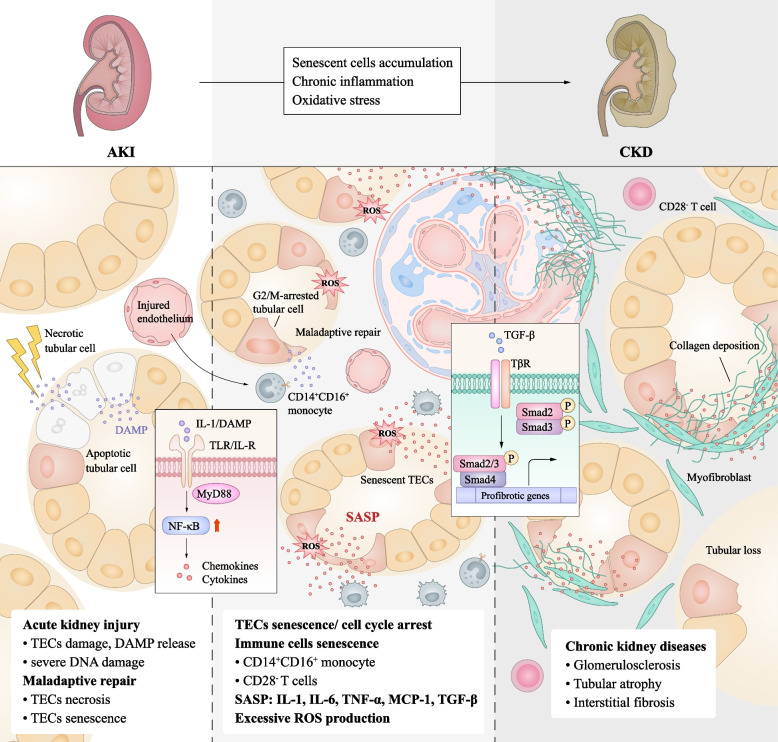
Senescent TECs accumulation after AKI drives the progression of CKD*.* Acute kidney injury induces cellular damage and DNA degradation in TECs. Maladaptive repair after AKI leads to TECs senescence. Senescent TECs accumulates in kidney with SASP, which mainly includes proinflammatory cytokines, growth factors, chemokines, and matrix remodeling enzymes. These proinflammatory and profibrotic molecules aggravate immune cells infiltration and tubular cell injury, leading to persistent tubulointerstitial inflammation, proliferation of fibroblasts, and excessive deposition of extracellular matrix, which leads to the exacerbation of renal injury and drives AKI to CKD. Senescent immune cells such as CD28^−^ T cells, CD14^+^CD16^+^ monocytes also aggravate chronic inflammation and ROS production in kidney, which promotes the progression of CKD

In the early phase of renal damage, tubular epithelial cells (TECs) senescence is extremely prevalent. A few days after kidney injury, TECs begin to senesce, which is regulated by Toll-like and interleukin 1 receptors (TLR/IL-1R) of the innate immune system [68]. Senescent TECs may cause senescence in surrounding cells in a paracrine manner; hence, the number of senescent cells increases over time [69]. The accumulation of senescent cells can directly deteriorate the microenvironment and contribute to tissue damage, premature renal aging, and increased susceptibility to chronic renal disease. Increased complement C5a binding to C5R on TECs in patients with coronavirus disease 2019 is responsible for TECs senescence and subsequent progression to CKD [70] (Fig. 2).

Tertiary lymphoid tissue develops in the kidneys of various mouse models following the kidney damage. The development of tertiary lymphoid tissue was a consequence of immunosenescence. It is also detectable in young patients with autoimmune disorders such as IgA nephropathy and anti-neutrophil cytoplasmic antibody vasculitis [71]. Tertiary lymphoid tissue spatially occupies a broad area of the renal parenchyma, induces intrarenal inflammation, and impedes intrinsic tissue repair. A substantial number of proinflammatory cytokines are generated in these tertiary lymphoid tissues, which may underlie chronic inflammation (inflammaging) in the kidney.

#### Immunosenescence in chronic kidney disease and end-stage renal disease

CKD describes structural and functional damage to the kidneys that persists over time and is accompanied by a persistent decrease in GFR. The CKD phases range from asymptomatic stage 1 to ESRD stage 5. From 1990 to 2017, the global prevalence of CKD increased by 29.3% (range: 26.4–32.6%), according to the Global Burden of Disease [72]. Statistically, more patients with CKD eventually develop ESRD and require renal replacement therapy, such as dialysis (43.1%) and kidney transplantation (34.4%) [73]. Treatment for advanced CKD is restricted and the prognosis is poor. Thus, early diagnosis and innovative therapeutics targeting these potential mechanisms are required. Morphological and functional changes in aging kidneys have been covered previously, including decreasing nephron loss, renal fibrosis, GFR reduction, tubule function decrease, and microvascular alteration. Senescent cell markers, senescence-associated galactosidase, p16, and Ki-67, are expressed at higher levels in the kidneys of aging mice and humans [74]. Owing to chronological aging, the older population is more susceptible to CKD. Further, patients with CKD and ESRD may also have premature immunosenescence, making them physiologically older than the general population. An analysis of T cells from patients with ESRD revealed premature immunological aging; the immunological age of patients with ESRD was 20–30 years older than that of healthy controls of the same chronological age [75].

Patients with CKD exhibit cellular alterations characterized by chronic inflammation, advanced cellular senescence, and immune system dysfunction. The long-term retention of uremic molecules and cytokines in patients with CKD leads to chronic inflammation. Premature aging of the immune system may be induced and accelerated in the persistent uremic milieu of patients with CKD [75, 76]. Additionally, oxidative stress, acidosis, infection, dysbiosis, and metabolic dysregulation may contribute to immunosenescence in patients with CKD and ESRD. Subsequently, exacerbated immunosenescence promotes disease development and increases the susceptibility to infection. Studies in pediatric CKD have shown that pediatric T-cell phenotypes were similar to those of the aging population in the chronic inflammation environment of CKD, including T cell exhaustion and senescence, naïve T cell reduction, and CD28 expression loss [77]. The CD14^+^CD16^+^ monocytes in patients with CKD were statistically higher than those in healthy controls, expressing higher levels of proinflammatory cytokines and vascular adhesion molecules than CD14^+^CD16^−^ monocytes. The high frequency of these cells facilitates the state of chronic inflammation, increases the risk of CKD progression to ESRD, and is associated with worse cardiovascular outcomes. Ironically, these changes seem to persist as even after successful kidney transplantation, the ratio of CD28^−^ T cells to CD14^+^CD16^+^ monocytes did not normalize inspite of reduction in the serum levels of proinflammatory cytokines [78, 79].

#### Immunosenescence in kidney replacement therapy

Over the past three decades, the number of patients over 75 years of age who developed ESRD has tripled from 7.6% to over 20% in the United States. As a result of population aging and the increased prevalence of age-related diseases, such as hypertension and diabetes mellitus, the elderly is the fastest-growing segment of patients diagnosed with ESRD, and millions of them die due to a lack of kidney replacement therapy (KRT). Dialysis, including peritoneal dialysis and hemodialysis, is a well-accepted KRT for irreversible renal failure. Kidney transplantation is the best health-preserving and economical KRT for ESRD; the number of global renal transplantation recipients with ESRD has increased by 34.4% from 1990 to 2017 [73]. As the demand for kidney transplants increases significantly, the average age of those on transplant waiting lists is also increasing. Therefore, to alleviate organ shortage, donor eligibility should be expanded to include elderly donors, resulting in more kidney transplants from older persons.

Despite improving the mortality risk of patients on dialysis over the past 50 years, mortality remains unacceptably high due to cardiovascular events and infection [80, 81]. Research found that the hallmark of immunosenescence, such as premature aging of CD4^+^ T cells, particularly the depletion of naïve T cells, was considered a strong predictor of mortality among patients on hemodialysis [82]. In addition, a 2018 study showed that atherosclerotic cardiovascular events were correlated with the circulating CD4^+^CD28^−^ T-cell population in patients on hemodialysis. Excess CD4^+^ T cells expressing this immunosenescence marker may significantly mediate atherosclerotic cardiovascular events in patients on dialysis [83]. In another study, the telomere attrition of mononuclear cells isolated from patients on hemodialysis was higher than that of age-matched controls, indicating that hemodialysis therapy might contribute to monocytic senescence [84]. In contrast, organs from elderly donors are more susceptible to ischemia/reperfusion injury, which enhances the release of DAMPs, resulting in a greater inflammatory response. Considering that older kidneys are more likely to be transplanted into older recipients, acute graft rejection would be more severe if it occurred; the intrinsic functional deficits and higher immunogenicity of the elderly kidney may be important factors.

Advanced age of organ transplant recipients or donors markedly affects renal transplantation outcomes. Retrospective studies have shown a higher acceptance rate of kidney transplantation in older recipients. In renal transplantation recipients older than 60 years, CD4^+^ T cell depletion is a common hallmark of immunosenescence and has been suggested to be linked to lower rates and weaker acute graft rejection [85, 86].

In addition, kidney transplantation may physiologically accelerate immunosenescence through inflammatory stimuli of alloantigen exposure. Innate Eomes^+^ CD8^+^ T cells are a unique subset that is closely associated with IFN-γ production during the innate response. In a pilot cohort of renal recipients, the number of innate Eomes^+^ CD8^+^ T cells was increased and exhibited an exacerbated senescent phenotype (CD27^−^, CD28^−^) [87]. Researchers have observed renal transplant recipients 1 year after transplantation. They found that their follow-up anti-thymocyte globulin (ATG) treatment had a toxic effect on thymic output and bone marrow-derived lymphoid progenitor cells. Terminally differentiated CD57^+^/CD28^−^ T cells are a senescent phenotype correlated with a higher risk of post-transplantation infection and acute rejection, which was found to expand in ATG-treated patients. In renal transplantation, follow-up immunosuppressive treatment [such as ATG and mycophenolate mofetil (MMF)] was associated with accelerated immunological aging after normalization of the uremic milieu with KRT [88]. The suppressive activity of regulatory T cells (Tregs) can be maintained in patients with ESRD by diverse differentiation pathways of inducible costimulatory (ICOS)^+^ and ICOS^−^ recent thymic emigrant-Tregs/responder T-cells [89]. However, this equilibrium is abrupt during long-term renal transplantations.

### Therapeutic strategy targeting the senescent immune system

Because of the pivotal role of senescent cells and the senescent immune system in age-related diseases, strategies to remove senescent cells or to block SASP have become imperative, and increasing interest has been aroused in rejuvenating the immune system. Senotherapeutics, including senomorphics and senolytics, can eliminate or delay adverse effects of cellular senescence and aging. Senomorphics are small molecules that dampen chronic inflammation in aging kidneys by blocking pathways related to SASP expression, such as p38 mitogen-activated protein kinase (MAPK) [90], NF-κB [91, 92], and silencing information regulator 2 related enzyme 1/SMAD3 [93]. Senomorphics may also inhibit senescence in neighboring cells by impeding the paracrine signaling of TECs. Senolytics are pharmacological agents that selectively kill senescent cells and impede their accumulation in tissues, consequently delaying senescence and improving age-related diseases. Senolytics diminish the population of senescent cells by interfering with senescent-cell anti-apoptotic pathways, including inhibition of the Bcl-2 family, phosphatidylinositol-3-kinase, thymidine kinases, and fork head box O4-p53 interaction [94]. The natural compound quercetin ameliorates kidney injury by inhibiting M1/M2 macrophage polarization, which is associated with downregulated activities of NF-κB p65 and IFN regulatory factor 5, thus leading to the inactivation of upstream signaling TLR4/Myd88 [95]. Dasatinib plus quercetin significantly decreased the senescent cell burden and renal inflammation in individuals with diabetic kidney disease [96]. The most effective strategy may be immune modulation as it activates the natural clearance of senescent cells, promotes renal tissue repair, and inhibits inflammation in senescent kidneys. T cell senescence plays a major role in age-related decline in immune function. p38 MAPK blockade can reverse senescence in CD8^+^ T cells by increasing their cellular proliferation, telomerase activity, and mitochondrial biogenesis via the mammalian target of rapamycin -independent pathway [97].

## Conclusion

With the steady increase in the number of elderly individuals globally, age-related diseases emerge as a major challenge to health care workers. Apart from functional and structural changes in the kidneys introduced by aging, immune system decline also significantly increases the risk of age-related kidney diseases. Immunosenescence is a loose definition of age-related changes in the innate and adaptive immune responses, which is characterized by shrinkage of naïve immune cell reservoirs, accumulation of late-stage differentiated cells with a senescent phenotype, and immunoglobulin class switching. These changes in the immune system result in two seemingly incompatible aspects: diminished immune response and increased inflammatory response, also known as inflammaging.

TECs senescence and tertiary lymphoid tissue formation occur following AKI. Senescent kidney cells promote a chronic inflammatory microenvironment, which can subsequently cause local tissue damage, hinder tissue repair, and promote immune system senescence. Intrarenal inflammation underlies the development of renal fibrosis and CKD later in life. Immunosenescence is exaggerated in patients with CKD and ESRD. Hallmarks of immunosenescence, including decreased naïve T cells, reduced CD28 expression, and increased proinflammatory macrophages, are convincing predictors of mortality in patients with CKD and ESRD. Renal replacement therapy for old patients with ESRD results in a lower acute rejection rate after the kidney transplantation. However, immunosenescence may increase the risk of chronic, but severe, graft failure. In addition, immunosenescence has been reported to speed up during kidney transplantation and immunosuppressive treatment (e.g., ATG and MMF).

In 1962, Walford first proposed the concept of immunity and aging. Numerous researchers have attempted to determine the relationship between immune aging and diseases that are common in the elderly, including for kidney diseases. However, most available studies (whether performed in humans or animals such as mice) are cross-sectional; ambiguous associations between the kidney disease and the senescent immune phenotype have been demonstrated in these cross-sectional studies. The immune system is shaped and undergoes changes throughout life. Longitudinal studies are required to elucidate the causal relationship between immunosenescence and kidney disease.

## Data Availability

Not applicable.

## Abbreviations

PD-1: Programmed death 1
CTLA-4: Cytotoxic T-lymphocyte-associated protein 4
CKD: Chronic kidney disease
GFR: Glomerular filtration rate
ESRD: End-stage renal disease
SASP: Senescence-associated secretory phenotype
IL: Interleukin
CXCL: Chemokine (C-X-C motif) ligand
NK: Natural killer
IFN: Interferon
TNF: Tumor necrosis factor
HSCs: Hematopoietic stem cells
LIR-1/ILT-2: Leukocyte immunoglobulin-like receptor-1/immunoglobulin-like transcript receptor-2
DCs: Dendritic cells
APC: Antigen-presenting cell
TLRs: Toll-like receptors
MHC: Major histocompatibility complex
mDC: Myeloid dendritic cell
pDC: Plasmacytoid dendritic cell
fDC: Follicular dendritic cell
DUSP6: Dual specific phosphatase 6
TCR: T cell receptor
HCMV: Human cytomegalovirus
TLRs: Toll-like and interleukin 1 receptors
NOD: Nucleotide-binding oligomerization domain
DAMPs: Damage-associated molecular patterns
PAMPs: Pathogen-associated molecular pattern
MAMPs: Metabolism-associated molecular patterns
AKI: Acute kidney injury
MyD88: Myeloid differentiation primary response protein 88
NF-κB: Nuclear factor-kappa B
ROS: Reactive oxygen species
TECs: Tubular epithelial cells; KRT, kidney replacement therapy
ATG: Antithymocyte globulin
MMF: Mycophenolate mofetil
Tregs: Regulatory T cells
ICOS: Inducible co-stimulatory
MAPK: Mitogen-activated protein kinase
ICB: Immune checkpoint blockade
PD-1: Programmed death 1
CTLA-4: Cytotoxic T-lymphocyte-associated protein 4
CAR: Chimeric Antigen Receptor
GFR: Glomerular filtration rate
TECs: Tubular epithelial cells
SASP: Senescence-associated secretory phenotype
DUSP6: Dual specific phosphatase 6
TCR: T cell receptor
NK: Natural killer
TNF: Tumor necrosis factor
HSCs: Hematopoietic stem cells
NCR: Natural cytotoxic receptors
APC: Antigen-presenting cell
MHC: Major histocompatibility complex
DCs: Dendritic cells
TLRs: Toll-like receptors
DAMPs: Damage-associated molecular patterns
PAMPs: Pathogen-associated molecular pattern
MAMPs: Metabolism-associated molecular patterns
TNF: Tumor necrosis factor
CRP: C-Reactive protein
AKI: Acute kidney injury
CKD: Chronic kidney disease
ESRD: End-stage renal disease
TECs: Tubular epithelial cells
TLR/IL-1R: Toll-like and interleukin 1 receptors
COVID-19: Coronavirus disease 2019
ANCA: Anti-neutrophil cytoplasmic antibodies
KRT: Kidney replacement therapy
IRI: Ischemia/reperfusion injury
ATG: Antithymocyte globulin
MMF: Mycophenolate mofetil
Tregs: Regulatory T cells
ICOS: Inducible co-stimulatory
RTE: Recent thymic emigrant
Tresps: Responder T-cells
SCAPs: Senescent-cell anti-apoptotic pathways.
